# The association of severe anemia, red blood cell transfusion and necrotizing enterocolitis in neonates

**DOI:** 10.1371/journal.pone.0254810

**Published:** 2021-07-20

**Authors:** Juan Song, Huimin Dong, Falin Xu, Yong Wang, Wendong Li, Zhenzhen Jue, Lele Wei, Yuyang Yue, Changlian Zhu

**Affiliations:** 1 Department of Neonatology, Henan Key Laboratory of Child Brain Injury and Henan Pediatrics Clinical Research Center, Third Affiliated Hospital and Institute of Neuroscience, Zhengzhou University, Zhengzhou, China; 2 Center for Brain Repair and Rehabilitation, Institute of Neuroscience and Physiology, Sahlgrenska Academy, University of Gothenburg, Gothenburg, Sweden; 3 Department of Women’s and Children’s Health, Karolinska Institutet, Stockholm, Sweden; Metrohealth Medical Center, UNITED STATES

## Abstract

**Background:**

The relationship between severe anemia, red blood cell transfusion and Neonatal necrotizing enterocolitis (NEC) remains controversial. The purpose of this study was to determine the association of severe anemia and RBC transfusion with NEC in neonates.

**Methods:**

The clinical characteristics of NEC were observed in 467 infants with different birth weights from January 2012 to July 2020. A 1:1 ratio case-control study was performed in very low birth weight (VLBW) infants. Severe anemia, RBC transfusion, and confounding factors, including maternal and perinatal complications, feeding, and antibiotics administration were collected in both groups. Univariate and multivariate analyses were used to investigate effects on the risk of NEC.

**Results:**

The day of NEC onset and mortality were inversely associated with birth weight. In VLBW infants, adjusting for other factors, severe anemia within 72 h [OR = 2.404, P = 0.016], RBC transfusion within 24 h [OR = 4.905, P = 0.016], within 48 h [OR = 5.587, P = 0.008], and within 72 h [OR = 2.858, P = 0.011] increased the risk of NEC.

**Conclusion:**

Both severe anemia and RBC transfusion appears to increase the risk of NEC in VLBW infants. The early prevention and treatment of anemia, strict evaluation of the indications for transfusion and enhanced monitoring after transfusion is encouraged in the NICU.

## Introduction

Neonatal necrotizing enterocolitis (NEC) is a common complication and leading cause of death in neonates, especially in very low birth weight (VLBW) infants with a mortality of 20–30% [1, 2]. At present, NEC is thought to develop often after exposure to non-breast milk, and due in part to increased reactivity of the premature intestinal mucosa to microbial ligands, which leads to mucosal injury and increased inflammation [3]. Newborns are at high risk of anemia in early life. More than half of VLBW infants require one or more RBC transfusions during hospitalization [4]. In recent years, some clinical studies have linked NEC with RBC transfusion in premature infants or VLBW infants [5–7]. About 5.2–35% of premature infants receive a transfusion within 24–72 hours prior to the onset of NEC [7–12]. However, other studies showed no association between RBC transfusion and NEC [13–15]. One retrospective cohort study even showed that RBC transfusion was a protective factor for NEC [16]. Some studies highlighted that severe anemia, not the transfusion event, was associated with the risk of developing NEC [17, 18]. Our previous study [19] indicated that erythropoietin treatment for severe anemia could decrease the incidence of NEC in preterm infants, which indirectly proves the association between severe anemia and NEC. Animal models suggested that anemia can induce intestinal inflammation and barrier disruption, and increase the risk for NEC [20, 21]. Thus, the relationship between RBC transfusion, anemia and NEC remains unclear. Therefore, we hypothesized that severe anemia or RBC transfusion could be a risk factor for NEC. To prove this hypothesis, we conducted a retrospective case-control study to analyse the association of severe anemia or RBC transfusion and the development of NEC in neonates.

## Materials and methods

### Study design and participants

This was a retrospective case-control study. From January 2012 to July 2020, neonates with different birth weights (<1500g, 1500-2499g, ≥2500g) who developed NEC stage II or III [22] in the neonatal intensive care unit were included. Infants with genetic or metabolic diseases, congenital abnormalities, meconium intestinal obstruction, spontaneous bowel perforations and Hirschsprung’s disease were excluded. Eligible controls were 1:1 matched for VLBW infants based on gestational age (± 3d), gender, and birth weight (± 200g) in the same admission month. The clinical manifestations of NEC were recorded. Severe anemia, RBC transfusion and confounding factors including maternal and perinatal complications, feeding, and antibiotics administration were recorded. Univariate analysis and multivariate analysis were used to investigate the relationship between severe anemia, RBC transfusion and NEC. All data were fully anonymized before collection, and were collected from May 2020 to October 2020 in the Third Affiliated Hospital of Zhengzhou University. The study protocol was approved by the Ethics Committee of The Third Affiliated Hospital of Zhengzhou University.

### Data collection

All data during the study period were collected from the medical record management database by experienced neonatologists. Clinical information including gestational age, birth weight, day of NEC onset, gestational age at day of NEC onset, clinical manifestations (abdominal distension, vomit, bloody stools, or apnea), intestinal perforation, surgery, and mortality of all infants with NEC were collected. For VLBW infants, basic information in the NEC group and control group including gestation age, birth weight, gender and delivery mode were collected. Hemoglobin (Hb) at birth, lowest hemoglobin, number and cumulative volume of RBC transfusions, and time between RBC transfusion and the day of NEC onset were collected. Meanwhile, confounding factors regarding maternal complications (such as gestational hypertension, gestational diabetes, premature rupture of membranes, placental abruption, and contamination of amniotic fluid), feeding mode and volume, pulmonary surfactant, umbilical or peripherally inserted central catheter (PICC) catheterization, antibiotics administration, mechanical ventilation, thrombocytopenia, patent ductus arteriosus (PDA), respiratory distress syndrome (RDS) and sepsis were collected in two groups. Medical care and management of the infants in the two groups were the same. In our study, all data were collected before the onset of NEC in the NEC group, while the timing point in the control group was determined using the day of diagnosis in the matched case; data were then collected before the reference point.

### Definitions

NEC was defined as Modified Bell stage ≥IIA NEC and required radiological evidence of pneumatosis, portal venous gas, or pneumoperitoneum in addition to clinical and laboratory features of NEC [22]. Abdominal X-ray score was used to evaluate the severity of abdominal symptoms according to Coursey [23]. Fulminant NEC was defined as NEC with rapid clinical progression with death or severe disease requiring surgical management occurring within 48 h of the onset [24]. Thrombocytopenia was defined by a platelet count of less than 100×10^9^/L in very premature infants, and a platelet count of less than 125×10^9^/L in late preterm or term infants [25].

Small for gestational age (SGA) was defined as a birth weight lower than the 10th percentile for gestational age. PDA was defined as continuous cardiac hemodynamic changes by cardiac ultrasound examination which requires pharmacological therapy or surgical ligation [26]. The diagnosis of sepsis included positive blood cultures and clinical sepsis. Infants with clinical sepsis had serious clinical infection symptoms and required advanced antibiotics or a combination of antibiotics [27]. Death was defined as an infant who died due to NEC.

### Severe anemia and RBC transfusion strategy

In full-term infants, severe anemia was defined as a hemoglobin concentration of less than 60 g/L [28]. In premature infants, severe anemia was determined based on the hemoglobin concentration, the days after birth and the respiratory status [29]. In more detail, less than 7 days after birth, the Hb of infants with respiratory support was less than 115g/L, and the Hb of infants without respiratory support was less than 110g/L. For infants within the second week of life, the Hb of infants with respiratory support was less than 100g/L, and the Hb of infants without respiratory support was less than 85g/L. For infants aged 3 weeks and older, Hb was less than 85g/L in infants with respiratory support and less than 75g/L in infants without respiratory support. Respiratory support was defined as an inspired oxygen requirement in excess of 25% or the need for a mechanical increase in airway pressure. RBC transfusion was determined for infants with severe anemia or moderate anemia with severe clinical manifestations [30] (S1 Table). Packed red blood cells were used. The blood was usually kept in a 4°C refrigerator in the Blood Transfusion Department, and the maximum storage time was 35 days. The infants fasted during the transfusion, which lasted for 4 hours.

### Statistical analysis

Data were analysed using SPSS 21.0 software (IBM, Armonk, NY). Quantitative data with a normal distribution were described as mean ± SD. Quantitative data with abnormal distribution were described as median (interquartile range). For univariate analysis, quantitative data with normal distribution or non-normal distribution were analysed using the *t test* or *Mann–Whitney U-test*. All count data were analysed using the *Chi-square test* or *Fisher’s exact test*. Multivariate logistic regression analysis was performed using the variables that were significant at a *P*-value <0.05. Separate models were performed for NEC and severe anemia; NEC and RBC transfusions. Combined models were performed to assess severe anemia and RBC transfusion, and interaction between them. A two-sided *P*-value < 0.05 was considered statistically significant.

## Results

### Study population and baseline information

Between January 2012 and July 2020, a total of 467 neonates with NEC were observed for clinical manifestations. For all infants with different birth weights, the day of NEC onset was 7.0 (4.0, 13.0) d. As birth weight increased, the day of NEC onset was progressively earlier (*P* < 0.05). In the VLBW infants, the birth weight was 1166 ± 203g, gestational age was 30.0 (28.9, 31.7) w, and gestational age at NEC onset was 32.5 (30.9, 34.2) w. Compared with infants with a birth weight of 1500-2499g and ≥2500g, the incidence of abdominal distension, apnea, thrombocytopenia, RBC transfusion and mortality was higher in VLBW infants (*P* < 0.05) (Table 1).

**Table 1:** Clinical manifestation of NEC in infants with different birth weight.

Variables		Birth weight			*P* value
	Total	<1500g	1500g-2499g	≥2500g	
	(n = 467)	(n = 166)	(n = 232)	(n = 69)	
The day of NEC onset, d	7.0 (4.0, 13.0)	13.0 (4.0, 24.0)	7.0 (4.0, 10.0)	3.0 (3.0, 5.5)	<0.001
Gestational age, w	33.4 (30.8, 35.4)	30.0 (28.9, 31.7)	34.1 (32.7, 35.4)	36.3 (35.7, 37.0)	<0.001
Gestational age at the day of NEC onset, w	34.9 (32.7, 36.4)	32.5 (30.9, 34.2)	35.1 (34.1, 36.4)	36.9 (36.4, 37.7)	<0.001
Birth weight, g	1814 ± 644	1166 ± 203	1948 ± 271	2921± 406	<0.001
Abdominal X-ray score	5 (3, 8)	6 (3, 8)	5 (3, 8)	6 (4, 8)	0.281
Fulminant NEC, n (%)	82 (17.56)	34 (20.48)	39 (16.81)	9 (13.04)	0.360
Abdominal distension, n (%)	391 (83.73)	153 (92.17)	185 (79.74)	53 (76.81)	0.001
Vomit, n (%)	119 (25.48)	38 (22.89)	66 (28.45)	15 (21.74)	0.338
Bloody stools, n (%)	254 (54.39)	50 (30.12)	152 (65.52)	52 (75.36)	<0.001
Apnea, n (%)	130 (27.84)	86 (51.81)	43 (18.53)	1 (1.45)	<0.001
Thrombocytopenia after NEC, ×10^9^/L, n (%)	96 (20.56)	50 (30.12)	39 (16.81)	7 (10.14)	<0.001
RBC transfusion, n (%)	59(12.63)	48(28.92)	9(3.88)	2(2.90)	<0.001
Perforation, n (%)	58 (12.42)	21 (12.65)	29 (12.50)	8 (11.59)	0.974
Surgery, n (%)	75 (16.06)	34 (20.48)	30 (12.93)	11 (15.94)	0.129
Death, n (%)	55 (11.78)	34 (20.48)	19 (8.19)	2 (2.90)	<0.001

### Maternal and neonatal clinical characteristics of the cases and controls in VLBW infants

Among the infants with NEC, a total of 59 (59/467, 12.63%) received RBC transfusion before NEC onset. Meanwhile, 81.36% (48/59) were VLBW infants, 15.25% (9/59) were infants with a birth weight of 1500-2499g, and 3.39% (2/59) were infants with a birth weight of ≥2500g. Therefore, we next focused on analysing the relationship between severe anemia, RBC transfusion and NEC in VLBW infants. The baseline parameters of infants with VLBW in the NEC group (n = 166) and control group (n = 166) were not significantly different (*P* > 0.05) (Table 2).

**Table 2:** Maternal and neonatal clinical characteristics of the cases and controls in very low birth weight infants.

Characteristics	Control	NEC	*P* value
	(n = 166)	(n = 166)	
Gestational age, w	30.3 ± 2.0	30.3 ± 2.1	0.916
Birth weight, g	1187 ± 191	1166 ± 203	0.334
Male, n (%)	91 (54.82)	91 (54.82)	1.000
Cesarean delivery, n (%)	130 (78.31)	134(80.72)	0.586
**Maternal characteristics**			
Hypertension of pregnancy, n (%)	75 (45.18)	71 (42.77)	0.658
Gestational diabetes, n (%)	25 (15.06)	20 (12.05)	0.423
Rupture of membranes, n (%)	53 (31.93)	46 (27.71)	0.401
Placental abruption, n (%)	6 (3.61)	18 (10.84)	0.011
Amniotic fluid contamination, n (%)	31 (18.67)	28 (16.87)	0.667
Mother’s age, y	31.2 ± 5.9	30.6 ± 4.8	0.330
Systole/diastole ratios (S/D) ≥ 3 ^a^, n (%)	54/107 (50.47)	60/127 (47.24)	0.623
** Neonatal characteristics**			
SGA, n (%)	80 (48.19)	94 (56.63)	0.124
Thrombocytopenia, ×10^9^/L, n (%)	30 (18.07)	36 (21.69)	0.409
Surfactant administration, n (%)	134 (80. 72)	138 (83.13)	0.568
Grade III-IV RDS, n (%)	41 (24.70)	33 (19.88)	0.291
Mechanical ventilation, n (%)	40 (24.10)	48 (28.92)	0.320
Umbilical or PICC catheterization, n (%)	81 (48.80)	107 (64.46)	0.004
PDA, n (%)	74/158 (46.84)	72/157 (45.85)	0.862
Antibiotics administration, n (%)	129 (77.71)	128 (77.11)	0.896
Antibiotics treatment time, d	5.0 (1.0, 11.0)	4.0 (1.0, 8.0)	0.179
Sepsis, n (%)	27 (16.27)	34 (20.48)	0.321
**Feedings**			
First feeding, d	3.0 (2.0, 3.0)	2.0 (2.0, 4.0)	0.094
Total feeding amounts, ml/d	56.0 (12.0, 129.0)	66.0 (0.0, 144.0)	0.791
Mixed milk, n (%)	115 (69.28)	105 (63.25)	0.246
Formula, n (%)	20 (12.05)	21 (12.65)	0.868
No feeds started, n (%)	31 (18.67)	40 (24.10)	0.228
**Variables associated with anemia and RBC transfusion**			
Hb at birth, g/L	163 ± 21	161 ± 26	0.412
Lowest Hb, g/L	114 (91, 147)	112 (89, 138)	0.086
Severe anemia, n (%)	30 (18.07)	39 (23.49)	0.223
Severe anemia within 72h of onset, n (%)	13 (7.83)	28 (16.87)	0.012
RBC transfusion, n (%)	34 (20.48)	48 (28.92)	0.075
Cumulative volume of RBC transfusions ^b^, ml/kg	35.3 ± 20.0	35.0 ± 20.0	0.945
Transfusion within 24h of onset, n (%)	3 (1.81)	14 (8.43)	0.006
Transfusion within 48h of onset, n (%)	3 (1.81)	16 (9.64)	0.002
Transfusion within 72h of onset, n (%)	9 (5.42)	24 (14.46)	0.006

Note: SGA, small for gestational age; RDS, respiratory distress syndrome; PICC, percutaneous intravenous central catheter; PDA, patent ductus arteriosus.

a: S/D refers to the ratio of systolic (S) and diastolic (D) velocity of the umbilical artery by umbilical arterial Doppler, and S/D ≥ 3 indicates intrauterine fetal distress.

b: Only include infants with red blood cell transfusion.

Thirty-one clinical variables, including maternal characteristics, neonatal characteristics, feedings, and variables associated with anemia and blood transfusion were used for univariate analysis. We found that cases had a higher incidence of severe anemia within 72 h (*P* < 0.05), and were more likely to be RBC transfused within 24, 48 and 72 h of NEC onset than controls (*P* < 0.05). In addition, the incidence of placental abruption (*P* < 0.05) and umbilical or PICC catheterization (*P* < 0.05) was significantly higher in infants with NEC compared to the controls (Table 2).

We further analyzed the association between anemia, blood transfusion and different degree of NEC and fulminant NEC. Univariate analysis showed that there was no association between anemia, blood transfusion with different degree of NEC or fulminant NEC (*P* > 0.05). However, the cumulative volume of RBC transfusions was significantly higher in infants with NEC stage III than NEC stage II (*P* = 0.034) (Table 3).

**Table 3:** Characteristics of different degree of NEC and fulminant NEC in very low birth weight infants.

Characteristic	NEC stage II	NEC stage III	*P* value	Non-fulminant NEC	Fulminant NEC	*P* value
	(n = 107)	(n = 59)		(n = 132)	(n = 34)	
Hb at birth, g/L	162 ± 27	160 ± 24	0.743	160 ± 27	164 ± 24	0.417
Lowest Hb, g/L	117 ± 31	109 ± 33	0.158	116 ± 32	108 ± 33	0.238
Severe anemia, n (%)	22 (20.56)	17 (28.81)	0.230	29 (21.97)	10 (29.41)	0.361
Severe anemia within 72h of onset, n (%)	14 (13.08)	14 (23.73)	0.080	19 (14.39)	9 (26.47)	0.094
RBC transfusion, n (%)	31 (28.97)	17 (28.81)	0.983	37 (28.03)	11 (32.4)	0.620
Cumulative volume of RBC transfusions ^a^, ml/kg	20.0 (18.3, 38.5)	44.4 (20.3, 58.3)	0.034	34.6 (19.3, 54.2)	21.2 (18.8, 44.4)	0.650
Transfusion within 24h of onset, n (%)	6 (5.61)	8 (13.56)	0.141	10 (7.58)	4 (11.76)	0.662
Transfusion within 48h of onset, n (%)	7 (6.54)	9 (15.25)	0.069	11 (8.33)	5 (14.71)	0.425
Transfusion within 72h of onset, n (%)	13 (12.15)	11(18.64)	0.255	18 (13.64)	6 (17.65)	0.749

Note: a: Only include infants with red blood cell transfusion.

### Univariate and multivariate logistic analysis for NEC in VLBW infants

In the multivariate logistic regression models adjusting for placental abruption and UVC/PICC, we found severe anemia within 72 h [OR = 2.404, *P* = 0.016], RBC transfusion within 24 h [OR = 4.905, *P* = 0.016], within 48 h [OR = 5.587, *P* = 0.008], and within 72 h [OR = 2.858, *P* = 0.011] increased the risk of NEC in VLBW infants (Table 4).

**Table 4:** Univariate and multivariate logistic analysis for NEC in very low birth weight infants.

Characteristic	Logistic model without covariates			Logistic model with covariates ^a^		
	OR	95%CI	*P* value	OR	95%CI	*P* value
Severe anemia within 72h of onset	2.388	1.190–4.794	0.014	2.404	1.180–4.897	0.016
Transfusion within 24h of onset	5.004	1.410–17.755	0.013	4.905	1.353–17.782	0.016
Transfusion within 48h of onset	5.796	1.656–20.287	0.006	5.587	1.568–19.907	0.008
Transfusion within 72h of onset	2.948	1.326–6.555	0.008	2.858	1.267–6.448	0.011

Note: a, multivariate logistic regression models adjusted for placental abruption and UVC/PICC.

In the combined multivariate models adjusting transfusion for severe anemia within 72 h, RBC transfusion within 24 h [OR = 3.775, *P* = 0.047], within 48 h [OR = 4.363, *P* = 0.026] and within 72 h [OR = 2.346, *P* = 0.047] continued to have an association with NEC (Table 5). There was no interaction between severe anemia within 72 h and RBC transfusion. In addition, in the combined multivariate models, placental abruption and umbilical or PICC catheterization increased the risk of NEC (*P* < 0.05).

**Table 5:** Logistic analysis for NEC for the combined effects of severe anemia and transfusion.

Characteristic	Logistic model without covariates ^a^			Logistic model with covariates ^b^		
	OR	95%CI	*P* value	OR	95%CI	*P* value
Transfusion within 24h of onset	4.032	1.107–14.621	0.035	3.775	1.015–14.041	0.047
Transfusion within 48h of onset	4.698	1.311–16.828	0.017	4.363	1.193–15.958	0.026
Transfusion within 72h of onset	2.465	1.082–5.615	0.032	2.346	1.012–5.437	0.047

Note: a, all models include both RBC transfusion and severe anemia within 72h.

b, logistic regression models adjusted for placental abruption, UVC/PICC.

## Discussion

NEC is one of the common complications with a high mortality in neonates, particularly in VLBW infants [1, 31]. In recent years, studies on the association of anemia, RBC transfusion and NEC have received extensive attention, but the association between them is still not clearly understood. In this case-control study, we found both severe anemia and RBC transfusion appears to increase the risk of the development of NEC in VLBW infants.

In our study, the average day of NEC onset was 13.0 (4.0, 24.0) d, and the average gestational age of NEC onset was 30.0 (28.9, 31.7) w in VLBW infants, which was in line with previous studies [5, 32]. We found severe anemia increased the risk of the development of NEC. Singh [18] conducted a case-control study on 111 preterm infants with NEC and 222 matched controls, and found that anemia was associated with increased odds of NEC. In addition, Patel [17] performed a multicenter cohort study in 598 VLBW infants and found that severe anemia but not RBC transfusion was associated with an increased risk of NEC. Our previous clinical trial found that the improvement of severe anemia reduced the incidence of NEC in preterm infants, which also suggested the impact of anemia on the development of NEC [19]. A prospective cohort study showed that anemia was associated with intestinal injury [33]. The possible mechanism is that anemia can reduce tight junction protein ZO-1 expression, increase intestinal barrier permeability, and increase intestinal inflammation through altered macrophage function, leading to intestinal injury that may increase the risk of NEC [20, 21].

Our study showed that the risk of NEC is increased in the subsequent 24 h to 72 h after RBC transfusion in VLBW infants, and the cumulative volume of RBC transfusions was higher in NEC stage III than NEC stage II. Multiple studies have shown that RBC transfusion may be associated with the increased risk of NEC, but this association appears to be temporal [18, 34]. Furthermore, the concept of transfusion-associated necrotizing enterocolitis had been proposed, which refers to the occurrence of NEC within 48 h after RBC transfusion in infants [35]. In a previous case-control study [7], among 3,652 infants, 49 cases developed NEC, and 17 occurred within 48 h after blood transfusion. They concluded that antecedent RBC transfusion within 48 h of NEC onset appears to be an independent risk factor for the development of NEC in premature infants. During processing and storage, the aging and decomposition of RBCs and inflammatory mediators can reduce the deformability of RBCs, increase their adhesion aggregation, and make it easy to cause microcirculation blockage in intestinal vessels after intravenous infusion [36]. Blood reperfusion of the superior mesenteric artery can easily disrupt the regulation of mesenteric blood circulation and increase susceptibility to intestinal barrier injury [37].

However, there were only 24 VLBW infants who received RBC transfusions in the 72 h prior to the onset of NEC in this study. The small sample size might not reach a substantial conclusion. Some studies also do not support a causal relationship between RBC transfusion and NEC [13, 16, 17]. RBC transfusion during evolving NEC and before NEC diagnosis may be a result of developing prodromal NEC itself, i.e. reverse causation [17]. Recently, two meta-analyses also showed insufficient evidence to support the possible association between transfusion and NEC, but both were of predominantly low-quality studies and showed high heterogeneity [14, 38]. Therefore, well designed prospective studies on this topic are needed.

Meanwhile, Our results suggested that placental abruption increased the risk of NEC in VLBW infants, which was consistent with an Australian study [39]. They analysed a cohort of 4649 preterm infants with a gestational age of 24–31 weeks; in total, 178 (3.8%) infants developed NEC, and multivariate analysis indicated that placental abruption was a risk factor for NEC [OR: 2.09, 95%CI (1.30–3.35)]. The possible mechanism is that maternal placental abruption inducing infant hypoxia, and insufficient blood flow to the viscera leading to intestinal hypoxia and ischemia [39]. We also found that umbilical or PICC catheterization increased the risk of NEC in VLBW infants. Endovascular catheters may destroy mesenteric blood circulation, which leads to intestinal ischemic injury [40]. However, the association between umbilical or PICC catheterization and NEC remains controversial [18, 40, 41].

There were some limitations of our study. First, the sample size of infants who developed severe anemia and received RBC transfusion within 72 h of onset was small. Thus, the conclusion needs to be better evaluated by expanding the sample size. Second, our study was performed in one center, which could lead to the inevitable selection bias. Third, this was a retrospective study, so it was hard to adjudicate NEC diagnoses and verify exactly when NEC was diagnosed. Meanwhile, some confounding variables such as feed regimes, feed status, hemodynamically significant PDA or hypotension were hard to evaluate precisely. Therefore, multicenter prospective trials are needed for further study.

## Conclusions

Both severe anemia and RBC transfusion appears to increase the risk of NEC in VLBW infants. Therefore, the early prevention and treatment of anemia, strict evaluation of the indications for transfusion and enhancing monitoring after transfusion is encouraged in the NICU. Prospective studies by expanding the sample size are needed to better assess the influence of anemia and transfusion on NEC.

## Supporting information

S1 Table Guidelines for blood transfusion in premature infants in China. (DOCX)
