# Supplementary material for: The association of severe anemia, red blood cell transfusion and necrotizing enterocolitis in neonates
Source: PLoS One. 2021 Jul 20;16(7):e0254810. doi: 10.1371/journal.pone.0254810 (PMC8291682; doi:10.1371/journal.pone.0254810)
Supplement: S1 Table — (DOCX) [file pone.0254810.s001.docx]

**S1 Table. Guidelines for blood transfusion in premature infants in China**

| **Hb (g/L)** | **Hct (%)** | **Mechanical ventilation** | **Symptoms of anemia** | **Blood infusion strategy** |
| --- | --- | --- | --- | --- |
| ≤110 | ≤35 | Moderate mechanical ventilation (MAP＞8cmH_2_O, FiO2＞40%) | - | 15ml/kg, PRBC 2-4 h |
| ≤100 | ≤30 | Mild mechanical ventilation (Any kinds of invasive mechanical ventilation or CPAP > 6cmH_2_O, FiO_2_ < 40% ) | - | 15ml/kg, PRBC 2-4 h |
| ≤80 | ≤25 | Oxygen supply, does not require mechanical ventilation | One of the following manifestations exists.   - Heart rate > 180 beats/min or breathing rate > 80 beats/min ( > 24 h) - Elevated lactic acid levels (≥2.5mmol/L), increased oxygen pressure ≥20% - Excessive energy demand (≥100kcal/kg/d) - Bradycardia or apnea (≥2 times within 24h) | 20ml/kg, PRBC 2-4 h (It can be divided into 2 infusions, 10ml/kg each time) |
| ≤70 | ≤20 | No clinical symptom | RET< 0.1×10^12^/L | 20ml/kg, PRBC 2-4 h (It can be divided into 2 infusions, 10ml/kg each time) |

Note: Hb, hemoglobin; Hct, hematocrit; PRBC, packed red blood cells; RET, Reticulocytes.
